# Focus on Alectinib and Competitor Compounds for Second-Line Therapy in *ALK*-Rearranged NSCLC

**DOI:** 10.3389/fmed.2016.00065

**Published:** 2016-11-30

**Authors:** Phu N. Tran, Samuel J. Klempner

**Affiliations:** ^1^Division of Hematology-Oncology, University of California Irvine, Orange, CA, USA; ^2^The Angeles Clinic and Research Institute, Los Angeles, CA, USA; ^3^Cedars-Sinai Medical Center, Samuel Oschin Comprehensive Cancer Institute, Los Angeles, CA, USA

**Keywords:** alectinib, NSCLC, ALK, second line, crizotinib, resistance

## Abstract

The management of anaplastic lymphoma kinase rearranged (ALK+) non-small cell lung cancer (NSCLC) exemplifies the potential of a precision medicine approach to cancer care. The ALK inhibitor crizotinib has led to improved outcomes in the first- and second-line setting; however, toxicities, intracranial activity, and acquired resistance necessitated the advent of later generation ALK inhibitors. A large portion of acquired resistance to ALK inhibitors is caused by secondary mutations in the ALK kinase domain. Alectinib is a second-generation ALK inhibitor capable of overcoming multiple crizotinib-resistant ALK mutations and has demonstrated improved outcomes after crizotinib failure. Favorable toxicity profile and improved intracranial activity have spurred ongoing front-line trials and comparisons to other ALK inhibitors. However, important questions regarding comparability to competitor compounds, acquired alectinib resistance, and ALK inhibitor sequencing remain. Here, we review the key clinical data supporting alectinib in the second-line therapy of ALK+ NSCLC and provide context in comparison to other ALK inhibitors in development.

## Background

Non-small cell lung cancer (NSCLC) accounts for 85% of all lung cancer and remains the leading cause cancer-related mortality in both men and women with a 5-year survival rate of less than 20% in US patients (1). Rapid advances in understanding the molecular pathogenesis of NSCLC have demonstrated that NSCLC is a heterogeneous group of diseases. Chromosomal rearrangements involving *ALK* and *ROS1* are present in 3–7% (2) and 2% (3) of patients with NSCLC, respectively. *ALK* translocations are found nearly exclusively in lung adenocarcinomas. Crizotinib, a first-generation ALK and ROS1 inhibitor, has resulted in improved progression-free survival (PFS) relative to chemotherapy in the first- and second-line settings for *ALK*-*rearranged (ALK*+*)* NSCLC. Compared to chemotherapy in treatment naïve *ALK*-rearranged patients, crizotinib led to higher objective response rate (ORR) (74 vs. 45%) and median PFS (10.9 vs. 7.0 months) but no difference in overall survival (hazard ratio for death with crizotinib, 0.82; 95% CI, 0.54–1.26; *P* = 0.36) (Table 1) (4). In *ALK*-rearranged patients with prior chemotherapy exposure, crizotinib also led to improved ORR (65 vs. 20%) and median PFS (7.7 vs. 3.3 months) (5). Like other oncogene driven tumors, acquired resistance is nearly universal in *ALK*+ NSCLC, and most develop crizotinib resistance within 1 year of treatment with central nervous system (CNS) metastasis being a major site of progression (6).

**Table 1:** **Comparison of second-line therapy trials in NSCLC**.

Compound	Phase	*n*	Study population	Primary endpoint	PFS	ORR	Reference
**ALK+ population**							
Ceritinib	I	246	ALK+ naïve and crizotinib failure	RP2D 750 mg qd	ALK inh naïve: 18.4 months	ALK inh naïve: 72%	(7)
					ALK inh expos: 6.9 months	ALK inh expos: 56%	
Alectinib	II	138	ALK+, crizotinib failure	ORR 50%	8.9 months	ORR 50%	(8)
						CNS DCR 83% among 84 pts with CNS mets	
Alectinib	II	87	ALK+, crizotinib failure	ORR 48%	8.1 months	ORR 48%	(9)
						CNS DCR 100% among 16 pts with CNS mets	
Alectinib	I/II	47	ALK+, crizotinib failure	ORR 55%	NA	Overall ORR 55%	(10)
						CNS ORR 52%	
Alectinib	I	46	ALK+ naïve	ORR 93.5%	NA	ORR 93.5%	(11)
Crizotinib vs. chemo	III	347	ALK+ prior chemo	PFS	7.7 vs. 3.0 months	65 vs. 20%	(5)
Crizotinib vs. chemo	III	343	ALK+ naïve	PFS	10.9 vs. 7 months	74 vs. 45%	(4)
					1 year survival rate 84 vs. 79%		
**Unselected population**							
Pembrolizumab vs. docetaxel	III	1,000	Unselected	OS: 12.7 vs. 8.5 months	4 vs. 4 months	18 vs. 9%	(12)
Nivolumab vs. docetaxel	III	272	SCC	OS: 9.2 vs. 6 months	1 year survival rate 42 vs. 24%	20 vs. 9%	(13)
Nivolumab vs. docetaxel	III	582	Non-SCC	OS: 12.2 vs. 9.4 months	1 year survival rate 51 vs. 39%	19 vs. 12%	(14)
Docetaxel + ramucirumab vs. docetaxel	III	1,253	Unselected pts after 1st line	OS: 10.5 vs. 9.1 months	4.5 vs. 3.0 months	23 vs. 14%	(15)
Erlotinib vs. docetaxel or pemetrexed	III	424	Unselected	OS: 5.3 vs. 5.5 months	1.4 vs. 2 months	NA	(16)
Pemetrexed vs. docetaxel	III	571	Unselected	OS: 9.3 vs. 8.0 months in non-squamous	2.9 months each arm	9.1 vs. 8.8%	(17)
				OS: 6.2 vs. 7.4 months in squamous			
Docetaxel vs. placebo	III	104	Unselected	OS: 7.5 vs. 4.6 months	10.6 vs. 6.7 weeks	7.1 vs. 0%	(18)

*Upper portion summarizes ALK+ trials and lower portion provides findings from key second-line chemotherapy and immunotherapy trials to provide context*.

While the propensity for intracranial failure on crizotinib is partly related to lower penetration of blood–brain barrier (19), systemic relapses are mediated by multiple mechanisms including secondary ALK mutations and compensatory bypass pathway activation. In nearly a third of patients, tumors have acquired secondary mutation in the ALK tyrosine kinase domain. The most common resistance mutation is the gatekeeper *L1196M* mutation, followed by the *G1269A* (20–22). Additional resistance mutations include *C1156Y, L1152R, G1202R, S1206Y, 1151Tins, F1174C*, and *D1203N*, among many others (Table 2) (23–25). These mutations blunt the efficacy of crizotinib by either increasing the ALK kinase affinity for adenosine triphosphate (ATP) (*G1269A* and *1151Tins*), inducing conformational change causing steric hindrance (*G1202R* and *S1206Y*) or interfering with the downstream signaling pathway (*L1152R*) (23). Amplification of the *ALK* fusion gene was observed either alone or in combination with other resistance mechanisms in both *in vitro* studies (20) and resistant clinical specimens (26). Beyond the *ALK* dominant resistance mechanism, preclinical work and progression biopsies from patients on ALK inhibitors have revealed crizotinib resistance from amplification of epidermal growth factor receptor (EGFR) pathway, insulin-like growth factor pathway (*IGF-1R*), *cKIT* mutation, and *SRC* activity (26–28).

**Table 2:** **Mutation coverage for ALK inhibitors in late stage clinical development**.

Mutations	Crizotinib	Alectinib	Certinib	Brigatinib	Lorlatinib	Reference
*EML4-ALK*	S	S	S	S	S	(29, 30)
*L1196M*	R	S	S	S	S	(21, 22, 24, 29–32)
*L1152P/R*	R	S	R	S	S	(22, 30–32)
*G1123S*	R	S	R	NA	NA	(30, 33)
*1151Tins*	R	S	R	NA	S	(22, 24, 30, 31)
*C1156Y*	R	S	R	S	S	(21, 22, 29–31)
*F1174V/C/L*	R	S	R	S	S	(22, 29–31, 34)
*I1171T/N/S*	R	R	S	NA	NA	(30, 32, 35)
*V1180L*	R	R	S	NA	NA	(35)
*G1202R*	R	R	R	S	S	(22, 24, 30, 31)
*G1269A/S*	R	S	S	S	S	(22, 30–32)
*F1245C*	R	NA	S	NA	NA	(30, 36)
*S1206C/Y/F*	R	S	S	R	S	(22, 24, 30–32)
*E1210K*	R	S	S	S	S	(30)
*L1198F*	S	R	R	S	R	(30, 37)
*D1203N*	R	S	S	S	S	(30)
*CMET amp*	S	R	R	R	R	(38)

*The letter S denotes mutations that are “sensitive” (clinical and/or preclinical data) to a given compound, and “R” denotes resistance. NA, data not available*.

While crizotinib ushered in a new paradigm for *ALK*+ NSCLC, the emergence of acquired resistance and rates of intracranial progression suggested ongoing clinical needs in *ALK*+ disease. The management of crizotinib failure has largely been informed by data from later generation ALK inhibitors including alectinib; however, other recent second-line trials outside *ALK*+ disease are worth brief contextual mention (Table 1). The phase III REVEL trial demonstrated that the addition of ramucirumab (a vascular endothelial growth factor receptor 2 monoclonal antibody) to docetaxel in unselected advanced NSCLC patients yielded higher response rate (23 vs. 14%), median PFS (4.5 vs. 3 months), and median OS (10.5 vs. 9.1 months) than docetaxel monotherapy (15). Similarly, in the phase III CheckMate 017 trial nivolumab yielded superior ORR (20 vs. 9%), median PFS (3.5 vs. 2.8 months), and median OS (9.2 vs. 6.0 months) compared with docetaxel in heavily pretreated unselected advanced squamous NSCLC patients (13). The CheckMate 057 trial found higher ORR (19 vs. 12%) and median OS (12.2 vs. 9.4 months) in patients with non-squamous NSCLC compared with docetaxel (14). The efficacy of pembrolizumab was demonstrated in phase II/III KEYNOTE-010 trial which compared pembrolizumab vs. docetaxel in more than 1,000 patients (12). Pembrolizumab led to improved median OS in the overall population (12.7 vs. 8.5 months). Among 442 patients with at least 50% PD-L1 expression, the median OS for the pembrolizumab 2 mg/kg, 10 mg/kg, and docetaxel groups was 14.9, 17.3, and 8.2 months, respectively.

## Alectinib Overview

The expanding appreciation of crizotinib-resistant ALK mutations spurred development of the second-generation ALK inhibitors. Alectinib is a potent and selective second-generation oral ALK inhibitor. Alectinib exhibits limited inhibitory activity against other protein kinases such as EGFR, fibroblast growth factor receptor 2 (FGFR2), human epidermal growth factor receptor 2 (HER2), hepatocyte growth factor receptor (MET), platelet-derived growth factor subunit B (PDGFB), and Janus kinase 1 (JAK1) (29). In cell free assays, the half maximal inhibitory concentration (IC_50_) of alectinib for enzyme activity of ALK was 1.9 nM and the dissociation constant (KD) value for ALK in an ATP-competitive manner was 2.4 nM (29). *In vitro* experiments demonstrated that alectinib induces caspase-mediated apoptosis in *EML4-ALK* cell lines and results in dose-dependent tumor growth inhibition (ED50 = 0.46 mg/kg) and regression in animal models (29). More importantly, alectinib displayed significant efficacy against crizotinib-resistant *ALK L1196M* (IC_50_, 2 nM) and G1269A (IC_50_, 9 nM) mutations (22, 29). Alectinib was also active against ALK C1156Y, F1174L, 1151Tins, and L1152R but not ALK G1202R (IC_50_, 70–80 nM) both *in vitro* and *in vivo* experiments (Table 2) (22).

## Alectininb for Crizotinib Failure

Clinical trials evaluating the safety and efficacy of alectinib have been conducted in Japan and the US as both first-line untreated and *ALK*+ patient progressing on crizotinib. Support for alectinib activity in crizotinib failure comes from the AF-002JG study in which alectinib at 300–900 mg BID was well tolerated, with the most common adverse events (AEs) being fatigue (30%), myalgia (17%), and peripheral edema (15%) (10). The recommended phase II dose was 600 mg BID. Of the 44 evaluable patients with crizotinib resistance, 24 (55%) patients had response, 16 (36%) had stable disease (SD), and 4 (9%) had progressive disease. Alectinib also demonstrated activity against CNS metastases in 21 patients with an intracranial response rate of 52% [29% complete response (CR), 24% partial response (PR), and 38% SD] (10). Similar results were seen in a North American trial of 87 patients with advanced ALK-rearranged NSCLC who were refractory to crizotinib (9). The ORR for alectinib was 48% with a median PFS of 8.1 months (95% CI, 6.2–12.6). Fifty two patients had brain metastases at enrollment and 21 (40%) patients experienced CNS tumor regression, including 13 (25%) patients who achieved CR. Alectinib 600 mg BID was well tolerated with predominantly low grade constipation (36%), fatigue (33%), myalgia (24%), and peripheral edema (23%). Finally, the large phase II global study (NP2873) examined the ORR of alectinib for crizotinib-refractory ALK+ patients (*n* = 138) (8). This study is notable for a high rate of CNS metastases (61%) at baseline. The ORR determined by independent review committee was 50% (95% CI, 41–59%) and the median PFS was 8.9 months (95% CI, 5.6–11.3). Alectinib was highly effective for CNS metastases, with ORR of 57% and DCR of 83%. Of the 23 patients with baseline untreated CNS metastases, 10 (43%) had a complete CNS response. The authors note that the cumulative CNS progression rate (24.8%) was lower than the cumulative non-CNS progression rate (33.2%), which suggests that alectinib may delay or prevent the emergence of CNS metastases. Alectinib 600 mg BID was well tolerated with common side effects including low grade constipation (33%), fatigue (26%), and peripheral edema (25%). Overall the similar response rate to alectinib between the US and Japanese patients indicate no ethnic difference in response. Additionally, there was no significant difference in alectinib exposure at 600 mg twice daily among a small subgroup of Caucasian and Asian patients who underwent pharmacokinetic analysis. Based on established activity, the Food and Drug Administration approved alectinib for the treatment of *ALK*+ NSCLC patients who progressed or were intolerant of crizotinib on December 11, 2015.

Based on promising second-line data and potential superiority over crizotinib, alectinib is being investigated in the first-line setting. In the phase I/II AF-001JP study conducted in Japan, patients with ALK inhibitor-naïve *ALK*+ NSCLC were treated with alectinib (11). Alectinib at 300 mg BID daily was well tolerated with few grade 3 toxicities or dose-limiting toxicities (DLTs) and ORR was observed in 43 out of 46 patients (93.5%) at this dose. On the other hand, the response rate for first-line crizotinib reported by Solomon et al. was 74% (4). Two phase III trials, ALEX (NCT02075840), and JapicCTI-132316, are currently comparing alectinib and crizotinib in ALK inhibitor-naive patients with *ALK*-rearranged NSCLC. Recently updated clinical data among 207 randomized patients in the J-ALEX trial were presented at the ASCO 2016 annual meeting (39). The primary endpoint was PFS and secondary endpoints included OS, ORR, CNS PFS, safety, and quality of life. In the alectinib arm, constipation (36%) was the only common event, while in the crizotinib arm nausea (74%), diarrhea (73%), vomiting (59%), visual disturbance (55%), dysgeusia (52%), constipation (46%), ALT elevation (32%), and AST elevation (31%) were seen in >30% patients. Alectinib was more tolerable than crizotinib with fewer grade 3/4 AEs (26.2 vs. 51.9%) which translated to a lower discontinuation rate (8.7 vs. 20.2%). The ORRs of the alectinib and crizotinib arms were 91.6 and 78.9%, respectively. The median PFS was not reached (CI, 20.3 to NR) but significantly higher than crizotinib 10.2 (CI, 8.2–12.0) with HR 0.34 (0.17–0.71). Complete data sets from first-line trials are eagerly awaited and may lead to additional indications for alectinib.

## Additional Second- and Third-Generation ALK Inhibitors

The second-generation ALK inhibitor ceritinib has *in vitro* activity against crizotinib-resistant mutations. Results from the open label multicenter ASCEND-1 trial showed that ceritinib yielded ORR of 72% (95% CI, 61–82) in 83 ALK inhibitor-naive patients and 56% (49–64) in 163 ALK inhibitor-resistant patients (7). Median PFS was 18.4 months in ALK inhibitor-naive patients and 6.9 months (5.6–8.7) in ALK inhibitor-pretreated patients. Among 94 patients with brain metastases, intracranial disease control was reported in 15 of 19 (79%) ALK inhibitor-naïve patients and in 49 of 75 (65%) ALK inhibitor-pretreated patients. In ALK inhibitor-resistant patients with CNS metastasis, the rates of intracranial CR, PR, and SD were 5, 13, and 47%, respectively. Common toxicities included diarrhea (80%), nausea (77%), vomiting (57%), fatigue (38%), abdominal pain (37%), decreased appetite (36%), constipation (30%), cough (29%), abdominal pain (23%), and dyspnea (21%). In April 2014, ceritinib 750 mg daily was approved by the US FDA for *ALK*+ previously treated with crizotinib.

Although both alectinib and ceritinib have shown promising systemic and CNS activity they are unlikely to be compared head to head in clinical trials. While ceritinib appears to have similar systemic response to alectinib, the intracranial response rate appears inferior to alectinib in crizotinib-resistant patients with CNS metastases. Accepting cross-trial comparison caveats the absolute median PFS is numerically shorter for ceritinib (6.9 months in the ASCEND-1 trial) than alectinib (8.9 months in the global NP2873 trial) in ALK inhibitor-resistant patients.

Other ALK inhibitors including brigatinib (*AP26113)* and lorlatinib (*PF-06463922*) have shown activity in crizotinib failure and highlight the non-overlapping resistance mutation coverage among current ALK inhibitors (Table 2). Briefly, brigatinib is a potent dual inhibitor of ALK and EGFR, including *ALK L1196M* and *EGFR T790M* mutants, shown in preclinical studies (40, 41). In the phase II ALTA study, 222 heavily pretreated *ALK*-rearranged patients were randomized to receive brigatinib 90 mg PO (arm A) vs. 180 mg PO qd (arm B) (42). The investigator-assessed ORRs of arm A and B patients were 46% (95% CI, 36–55%) and 54% (95% CI, 44–63%), respectively. Median PFS in arms A and B was 8.8 and 11.1 months, respectively. However, the median follow-up was only 8.3 months and longer follow-up is needed to confirm the higher PFS observed in arm B. Among patients with active brain metastases at baseline, intracranial ORRs, as assessed by independent review committee, in A and B were 37% (7/19) and 73% (11/15), respectively. Most common AEs in arms A/B included nausea (33/40%), diarrhea (19/38%), headache (28/27%), cough (18/34%), dyspnea (21/21%), fatigue (20/27%), constipation (19/15%), abdominal pain (17/8%), and vomiting (24/23%). Grade ≥ 3 treatment-emergent AEs (A/B) included: increased CPK (3/8%), hypertension (4/5%), pneumonia (3/5%), rash (1/4%), and pneumonitis (2/3%). Discontinuations and dose reductions due to AEs (A/B) were 3/6% and 7/18%, respectively. Due to the favorable efficacy and toxicity profile, brigatinib 180 mg PO daily was chosen as the optimal dose and is moving forward in the phase III ALTA-1L vs. crizotinib in the first-line setting.

Lorlatinib (PF-06463922) is a third-generation reversible, potent ATP-competitive small molecule, inhibitor of ALK and ROS1. Lorlatinib has demonstrated activity against the majority of known resistant ALK mutations, except for *L1198F* (Table 2) (31, 37). Early data from an ongoing phase I/II study of lorlatinib in mostly pretreated patients with *ALK*+ and *ROS1*+ NSCLC were presented at the ASCO 2016 annual meeting (43). Among the 54 evaluable patients who received dose escalation from 10 mg to 200 mg, the overall response rate was 50% and intracranial response rate was 44% for target and non-target lesions and 60% for target lesions. The most common treatment-related AEs were hypercholesterolemia (54%) and peripheral edema (37%). Hypercholesterolemia was the most common (9%) grade (G) ≥ 3 treatment-related AE and most frequent reason for dose delay/reduction. No patient was discontinued due to a treatment-related AEs. The phase II dose was identified as 100 mg once daily. Pharmacokinetic analysis of four patients revealed that the unbound CSF to plasma drug ratio ranged from 0.61 to 0.96, indicative of good CSF penetration. In contrast, the ratio of CNS to serum concentration of crizotinib has been in the range of 0.0006–0.001 in previous reports (19, 44). Lorlatinib is effective against the *G1202R* mutation (Table 2).

## Conclusion/Future Directions

Over the past decade, there has been a remarkable progress in the target therapy for the management of *ALK*-rearranged NSCLC. Second- and third-generation inhibitors demonstrate broader coverage against crizotinib-resistant *ALK* mutations and often more favorable side effect profiles. As discussed elsewhere in this issue, we are approaching a paradigm in which understanding the exact resistance mechanism will inform the optimal choice and perhaps sequencing of ALK inhibitors. The approval of alectinib for crizotinib failure highlights major areas of focus in *ALK*+ disease; toxicity profile, intracranial activity, and resistance mutation coverage. While alectinib compares favorably in these areas, ongoing results from first-line trials and direct comparison against current and emerging ALK inhibitors will be important to refine optimal alectinib usage. Here we have provided a review of the clinical data supporting the activity of alectinib in the management of *ALK*+ NSCLC with a focus on the second-line setting in advanced disease.

## Author Contributions

PT and SK are involved in the conception/design and drafting the manuscript. All the authors approved the final version of the manuscript and agreed to be accountable for all aspects of the work in ensuring that questions related to the accuracy or integrity of any part of the manuscript.

## Conflict of Interest Statement

The authors declare that the research was conducted in the absence of any commercial or financial relationships that could be construed as a potential conflict of interest. The reviewer GP and handling Editor declared their shared affiliation, and the handling Editor states that the process nevertheless met the standards of a fair and objective review.
